# Predator–prey interactions in the canopy

**DOI:** 10.1002/ece3.6518

**Published:** 2020-07-29

**Authors:** Mark A. Linnell, Damon B. Lesmeister

**Affiliations:** ^1^ U.S. Forest Service, Pacific Northwest Research Station Corvallis OR USA; ^2^ Department of Fisheries and Wildlife Oregon State University Corvallis OR USA

**Keywords:** arboreal ecology, bin width for continuous data, camera‐trap monitoring, foraging mode, predator–prey interactions

## Abstract

Small mammal abundances are frequently limited by resource availability, but predators can exert strong lethal (mortality) and nonlethal (e.g., nest abandonment) limitations. Artificially increasing resource availability for uncommon small mammals provides a unique opportunity to examine predator–prey interactions. We used remote cameras to monitor 168 nest platforms placed in the live tree canopy (*n* = 23 young forest stands), primarily for arboreal red tree voles (tree voles; *Arborimus longicaudus*), over 3 years (*n* = 15,510 monitoring‐weeks). Tree voles frequently built nests and were detected 37% of monitoring‐weeks, whereas flying squirrels (*Glaucomys oregonensis*) built nests infrequently but were detected 45% of monitoring‐weeks. Most nest predators were detected infrequently (<1% of monitoring‐weeks) and were positively correlated with tree vole presence. Weasels (*Mustela* spp.) were highly effective predators of tree voles (*n* = 8 mortalities; 10% of detections) compared to owls (*n* = 1), flying squirrels (*n* = 2), and Steller's jays (*n* = 1). Tree vole activity decreased from 84.1 (95% confidence interval [*CI*]: 56.2, 111.9) detections/week 1‐week prior to a weasel detection to 4.7 detections/week (95% *CI*: 1.7, 7.8) 1‐week postdetection and remained low for at least 12 weeks. Interpretations of predator–prey interactions were highly sensitive to how we binned continuously collected data and model results from our finest bin width were biologically counter‐intuitive. Average annual survival of female tree voles was consistent with a previous study (0.14; 95% *CI*: −0.04 [0.01], 0.32) and high compared to many terrestrial voles. The relative infrequency of weasel detections and inefficiency of other predators did not provide strong support for the hypothesis that predation per se limited populations. Rather, predation pressure, by reducing occupancy of already scarce nest sites through mortality and nest abandonment, may contribute to long‐term local instability of tree vole populations in young forests. Additional monitoring would be needed to assess this hypothesis.

## Graphical abstract

In a multi‐year study, manipulative study, we observed strong responses of prey to additions of artificial nest platforms in the tree canopy. This was followed by a differential response based on foraging mode by their predators in year 3 with active, seeking predators exhibiting the strongest interactions with prey at nest platforms. We concluded that foraging mode can mediate predator–prey interactions in the tree canopy.

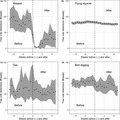

## 1 INTRODUCTION

Lack of resources, including availability of nest substrates and food, can limit abundances of small mammals (Hanski, Hansson, & Henttonen, 1991; Ransome & Sullivan, 2003). In turn, predators are frequently limited by prey availability and high abundances of small mammalian prey, at least locally, can support increased predator numbers albeit temporally lagged behind prey abundances (Hanski et al., 1991). Artificially increasing resources, including structural habitat (e.g., nest boxes), can provide a unique opportunity to examine intrinsic increases in abundances of the targeted population but also potential responses of the broader vertebrate community, including predators (Aitken & Martin, 2012; Dunn, 1977; Le Roux et al., 2016). Yet, studies of wild small mammal (<1 kg) population response to increases in structural habitat and the potential responses of predators and competitors remain relatively scarce (Newton, 1994).

Predators can reduce abundances of their prey lethally and by inducing nonlethal behavioral constraints to foraging, resting, and reproduction (Brown & Kotler, 2004; Preisser, Bolnick, & Benard, 2005). Behavioral responses of prey (Mäkeläinen, Trebatická, Sundell, & Ylönen, 2014) can depend on predator foraging mode. Prey species can use scent cues to avoid locations, including their nests, where active seeking predators (e.g., weasels, *Mustela* spp.; King & Powell, 2007) have visited or rely on environmental cues, such as low light intensity and extensive overhead cover, to reduce risk from sedentary ambush predators, such as most forest owls, while foraging (Jaksić & Carothers, 1985; Jędrzejewski & Jędrzejewska, 1990; Jędrzejewski, Rychlik, & Jędrzejewska, 1993; Kotler, Brown, & Hasson, 1991). Prey vulnerability to predators exhibiting different foraging modes may thus vary depending on where encounters occur, whether at prey resting or foraging sites.

Statistical models used to interpret the timing and extent of biological phenomena can be sensitive to the period in which observations are made (Steenweg, Hebblewhite, Whittington, Lukacs, & McKelvey, 2018). Continuous monitoring, such as remote camera or video devices, enables varying the temporal grain of observation (e.g., time bin width of 1‐hr vs. 1‐day), providing insights into statistical sensitivity but also potentially to the temporal aspects of a biological phenomenon. The decision to bin continuous data to a coarser temporal grain, however, is often arbitrary or based on properties of statistical models rather than observed biological phenomena (Sollmann, 2018).

In 2015, Linnell, Lesmeister, Bailey, Forsman, and Swingle (2018) initiated a study examining the response of arboreal rodents (red tree vole, *Arborimus longicaudus*; Humboldt flying squirrel, *Glaucomys oregonensis*; Douglas' squirrel, *Tamiasciurius douglasii*) to an increase in nest substrates in young forests (<80 years old), a resource hypothesized to be limiting there as compared to old forests (≥80 years old). They observed a 5.8‐fold increase (95% confidence interval [*CI*]: 2.9, 9.2) in plot‐level occupancy of the main target population (red tree voles, henceforth: tree voles), a small arboreal rodent that builds nests and forages exclusively in the live tree canopy, and that was not likely to be limited by food as their diet primarily consists of conifer needles, which are readily available in conifer forests. Tree voles are important prey for predators that exhibit different foraging modes, including forest owls (northern spotted owl, *Strix occidentalis caurina*; barred owl, *Strix varia*; northern saw‐whet owl, *Aegolius acadicus*) and weasels (*Mustela erminea, Mustela frenata*), and have an annual survival of 0.15 (95% *CI*: 0.06, 0.31) with most mortality attributed to predation (Forsman, Anthony, & Zabel, 2004; Forsman & Maser, 1970; Swingle, Forsman, & Anthony, 2010; Wiens, Anthony, & Forsman, 2014).

Herein, we describe predation and nonlethal avoidance of the suite of nest predators and competitors of arboreal rodents, in particular tree voles, during monitoring of artificial nest substrates (henceforth, nest platforms) over 3 years. We describe the lethal and nonlethal short (i.e., ~1 week) and long‐term effects (12 weeks) of four taxa that are documented predators of tree voles (weasels, owls) or that may exhibit competition but represent low predation risk (flying squirrels, probing, or digging birds) on patterns of nest occupancy by tree voles as observed by remote cameras placed directly above nest platforms (Graham & Mires, 2005; Swingle et al., 2010). We predicted that weasels and forest owls would have an immediate lethal effect (mortality) and cause longer term nonlethal avoidance of nest platforms. Interactions between Humboldt flying squirrels (henceforth, flying squirrels) and tree voles, as estimated from nest platform occupancy, are likely to be more subtle involving weak correlations although have the potential to influence nest occupancy if those interactions occur frequently.

## 2 MATERIALS AND METHODS

### 2.1 Study area

**FIGURE 1:**
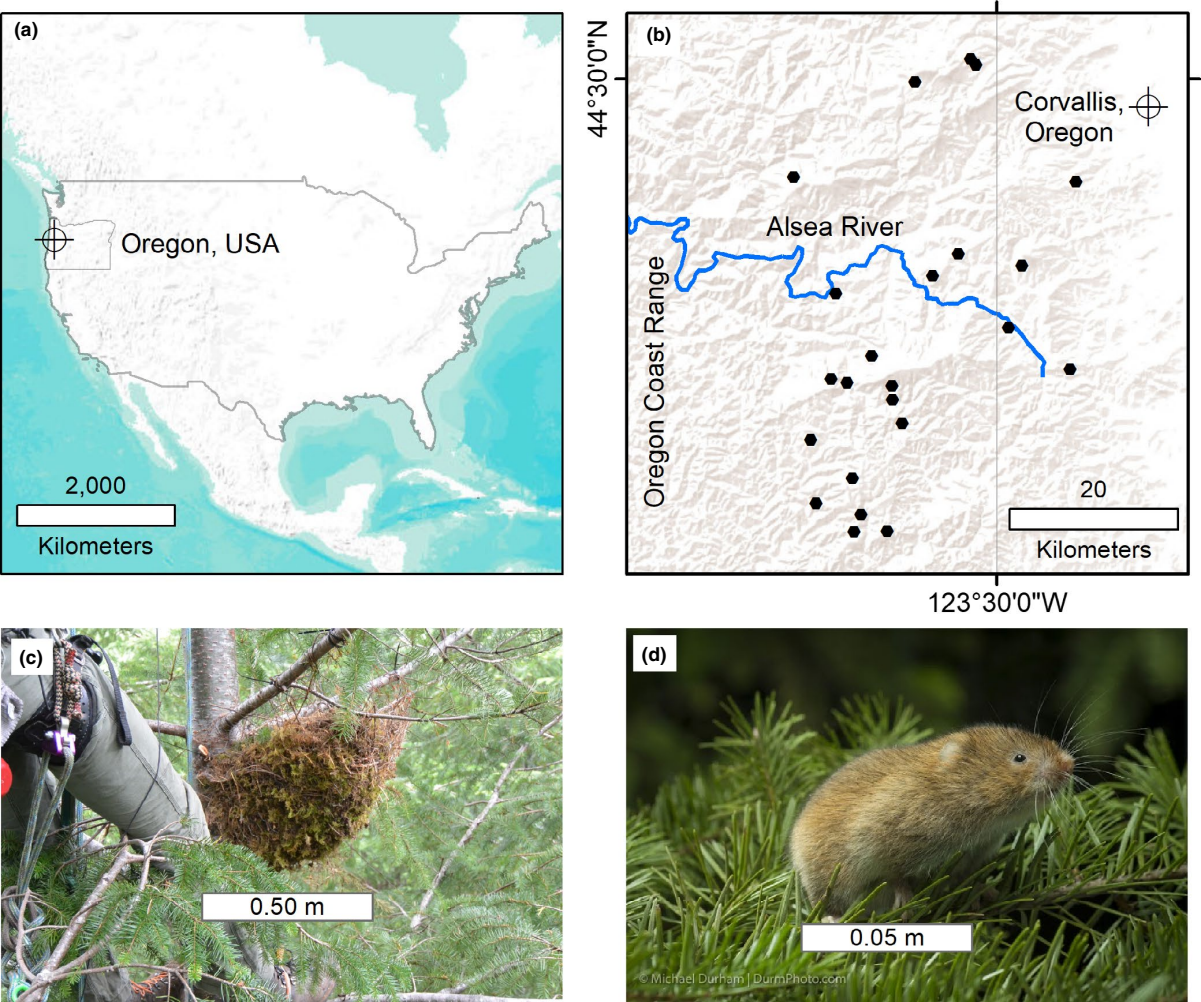
The scale at which the study was conducted: (a, b) The study area was located on the eastern edge of the central Coast Range in western Oregon, USA, at approximately 44°30′0″N 123°30′0″W, (c) we placed 2 nest platforms per ha at 23 randomly located young forest sites. Nest platforms were designed to provide nesting substrates for our primary target species, the red tree vole (d; photo courtesy of Michael Durham)

We conducted this study on federal forest lands in the eastern portion of the central Oregon Coast Range (44°30′0″N 123°30′0″W; Figure 1). Vegetation was primarily conifer forests dominated by Douglas‐fir (*Psuedotsuga menziesii*) and western hemlock (*Tsuga heterophylla*), growing on steep terrain with numerous and deeply incised drainages. The climate was cool and wet during winter (i.e., wet season; November 1–March 31) with occasional sub‐freezing temperatures and snow, and warm and dry in the summer (i.e., dry season; April 1–October 31). Forest age was highly correlated with management history, fire, and land ownership. Old forests (>80 years old) were located primarily on federal lands in relatively small patches in a matrix of young forests (<80 years old) and nonforest cover types (Kennedy & Spies, 2004; Linnell, Davis, Lesmeister, & Swingle, 2017; Wimberly & Ohmann, 2004). Young forests (22–44 years old) in this study were typical of the region and were established as conifer plantations, which resulted in stands dominated by Douglas‐fir trees with straight‐boles, simple branches, few cavities, and highly interconnected live crowns.

### 2.2 Site selection and monitoring of nest platforms

We added 598 nest platforms at a height of 16 ± 4 m ($\bar{x}$ ± 1 standard deviation [*SD*]) to 23 randomly selected young forest sites (17 in 2015, six in 2016) that were located adjacent to old forests that contained sign of tree vole presence (Linnell et al., 2018). At each site, we randomly selected two 100 m^2^ circular plots per hectare and constructed 1 nest platform in the tree at plot center within the live canopy (live limbs vertically above and below). To construct a nest platform, we stretched a length of hexagonal wire mesh (2.54 cm openings) between two or three branches to form an open basket and placed ~8 L of conifer branch tips and moss within the basket.

We used two sources of data: annual nest platform inspections (*n* = 1,640) that occurred each summer 2016–2018, and photographic data from nest platforms monitored with a remote camera (*n* = 168) June 2015–October 2018. Each nest platform was inspected annually for diagnostic sign of arboreal rodent nests. Tree vole nests, especially female nests, are frequently large (0.06 m^3^) and consisted of tunnels and nest chambers formed within discarded resin ducts, fecal pellets, and conifer branch tips whereas flying squirrels primarily built smaller cup‐shaped nests from collected moss (Lesmeister & Swingle, 2017; Swingle, 2005). Tree vole nests were inhabited primarily by one adult except for breeding female nests which often contained juveniles (Swingle, 2005).

We deployed remote cameras at a random selection of ~10% (2015) or ~20% (2016) of nest platforms (*n* = 96). In addition to the randomly selected nest platforms, we placed cameras at 72 nest platforms built in 2015 and containing a tree vole nest identified during the first‐year annual inspection in 2016. We combined data collected from randomly (*n* = 96) and nonrandomly (*n* = 72) placed cameras for all analyses (*n* = 168 monitored nest platforms).

Remote cameras were mounted 0.6–1.0 m above nest platforms and faced down such that the entire nest platform and some adjacent branches were within the field of view of the camera sensor and included in each image. We set each camera to record photographs when triggered by motion with a 5‐min (2015–2016) or 1‐min (2016–2018) quiet period. We tagged each photograph with species identity, and for tree voles, we also identified age class (juvenile, adult) and, if present, we noted the unique external marking on the tree vole. We tracked photograph tagging and estimated a rate of 2,616 photographs per‐hour (95% *CI*: 2,406, 2,826; *n* = 175 sessions). Using this rate, we estimated that tagging the 852,000 photographs in our data set required 326 hr (95% *CI*: 301, 354).

### 2.3 Binning of continuously collected remote camera data

Observations of spatial and temporal activity patterns can potentially provide insights into predator–prey relationships as animals perceive their environment through time and space (Hut, Kronfeld‐Schor, van der Vinne, & De la Iglesia, 2012). Because species interactions are often complex and difficult to identify, we used photographic detection (1) or nondetection (0) data to examine temporal overlap of tree voles and potential predators at 1‐hr, 1‐day, and 1‐week time bin widths. Potential nest predators were owls, weasels, or probing/digging birds with flying squirrels modeled as both prey and predator, depending on the model. We used a 1‐month bin width to quantify multi‐annual trends of activity at nest platforms.

### 2.4 Predator–prey temporal overlap models

We fit nine logistic regression models using tree vole as the dependent variable (three time bin widths × three time lags [described below]) and six models using flying squirrels. In each model, dependent (tree vole or flying squirrel) and independent (nest predators) variables were binomially distributed detection (1) or nondetection (0) data. At time lag *t*0 (unlagged), we examined whether nest predator presence was correlated with tree vole presence. Tree voles used nest platforms intensively with a high number of detections per‐day (Linnell et al., 2018); therefore, we interpreted positive correlations as representing potential attraction of the nest predator to the nest while tree voles were present.

To examine hypotheses of lagged effects of predator presence on tree vole presence (*t* + 1, *t* + 12), we used predator detection in the previous time step as the independent variables in our *t *+ 1 and *t* + 12 models. For example, a weasel detected on occasion three in a *t*0 encounter history of five occasions (00100) would result in a *t* + 1 of 00010. A negative correlation at *t* + 1 (whereby a tree vole or flying squirrel was absent (0) at *t* + 1) was interpreted as potentially arising from the nest predator detection (1) at *t*0. To examine a potential longer term, up to 12‐week lag in tree vole response to predator detections, we developed an independent variable for any previous detection of a predator during the encounter history. For example, a predator detected during time three of a 15 occasion encounter history with a t0 of (001000000000000) would result in 000111111111111 for model *t* + 12. Tree vole models included time lags of *t*0, *t* + 1, *t* + 12, and flying squirrel models time lags of *t*0 and *t* + 1.

We made several a priori predictions of positive (+), negative (−), or neutral correlations (=) with the number of symbols indicating the strength of the predicted relationship. We predicted that weasels and owls would be attracted to occupied tree vole nests at *t*0 (++), that these predators would negatively affect occupancy at *t* + 1 if tree voles were killed or avoided nests after a predator detection (−−−) but that nests would be re‐occupied over a longer time period, weakening negative correlations (*t* + 12; −). We predicted that flying squirrels would be weakly attracted at *t*0 (+) as they potentially use the same nests as tree voles and that those effects would remain weak through time at *t* + 1 (+) and *t* + 12 (+). Birds digging would be weakly positively correlated at *t*0 (+) as they would be targeting insects in decaying organic materials prevalent at tree vole nests but that co‐occurrence would be incidental with no effect through time at *t* + 1 (=) and *t* + 12 (=). For flying squirrels as the dependent variable, we predicted similar correlational trends as tree voles with owls and weasels negatively correlated (*t*0 = +; *t* + 1 = −) but that strength would be moderate (Linnell et al., 2018). We predicted no interactions with digging birds (=).

We used generalized linear mixed models with a logistic link function for our analyses. To account for spatial and temporal dependence of observations, we modeled individual nest platforms and the next coarser bin width (e.g., 1‐week bin width for 1‐day bin width encounter histories) as random effects. We represented a priori hypotheses as fixed effect independent variables (R package MCMCglmm; Hadfield, 2010; R Core Team, 2018). We used uniform and multivariate normal priors for fixed and random effects parameters and used the inverse‐Wishart distribution for variance components of priors. We used four Markov chains of 200,000 with a burn‐in period of 100,000, and set the thin to 0.02. To assess convergence, we visually evaluated chains and estimated the Gelman–Rubin convergence diagnostic in the coda package in R (Brooks & Gelman, 1998; Gelman et al., 2014; Plummer, Best, Cowles, & Vines, 2006; R Core Team, 2018). We used values of convergence diagnostics for parameters with <1.1 indicating chain convergence. We reported means and 95% credible intervals [*CrI*] of the posterior distributions, and interpreted log odds coefficients as probability of presence. In addition, we transformed some model output to odds ratios for ease of interpretation.

### 2.5 Lethal and nonlethal effects of predators and temporal trends in activity

We summarized observed cause‐specific mortalities of tree voles by recording when a dead tree vole was observed in the presence of a predator on the nest platform. We interpreted these events as the strongest causal evidence of mortality of tree voles by a specific nest predator. In addition, we used a qualitative likelihood of weasel predation, whereby we suspected that weasel predation went unobserved, to summarize activity patterns where a predation event was high (or observed) compared to low (Appendix A).

We examined nonlethal effects of nest predators on tree vole activity (No. of detections per week) 12 weeks before and 12 weeks after a predator was detected. We were interested in nonlethal effects per se but could not disentangle nest abandonment from mortality and so excluded zeros (weeks in which no tree vole was detected at a given nest platform) resulting in sample size differences for each week. This almost certainly decreased our sensitivity to detect changes attributable to avoidance behavior that resulted in no detections (zeros) and so we interpreted decreases in activity as strong evidence of nonlethal effects attributable to predators.

Predators can adjust their temporal activity patterns to coincide with those of their main prey (Forsman, Anthony, Charles Meslow, & Zabel, 2004; Forsman, Anthony, & Zabel, 2004). To examine whether temporal activity of predators coincided with those of their prey, we created two density plots using a 1‐hr bin width (i.e., only one detection per‐hour per‐day was used). First, we examined diel overlap of tree voles, flying squirrels, owls, weasels, and digging birds. Second, we examined activity patterns of high and low likelihood of predation by weasels. Finally, we assessed seasonal and multi‐annual trends of occupancy (proportion of monitored nest platforms occupied by month).

### 2.6 Apparent survival of tree voles

We estimated annual apparent survival for tree voles using a Cormack‐Jolly‐Seber model implemented in R package RMark (Laake 2013; White & Burnham, 1999). During annual nest platform checks, we attempted to capture any tree voles that were present. We externally marked each captured tree vole (marked tree vole) by clipping a 20 mm square from the tips of their dorsal fur in part of a quarter section of their back (e.g., “top‐right”) such that each tree vole within a site was uniquely marked. We resighted those individuals and analyzed 1‐day bin width encounter histories of marked tree voles that began on the date of capture. Capture and handling methods were approved by the U.S. Forest Service Institutional Animal Care and Use permit #2016‐009 and Oregon Department of Fish and Wildlife Scientific Taking Permit 041‐18.

Because tree voles molt growing their fur at unknown intervals and we had only one capture occasion per year, we right‐censored encounter histories to a survey period such that we minimized the uncertainty that animals were likely to leave the sample due to their mark fading while also retaining most of the data. To estimate the longevity of marks, we reviewed sequential photographs of marked female tree voles (*n* = 27) for which we reasonably certain that the marked tree vole remained at the nest but that the mark faded to became indistinguishable. We estimated that 20% of marks became indistinguishable at 63 days or fewer although some lasted longer as we estimated marks faded at a median of 88 days and a mean of 83.4 ± 28.7 days (*n* = 20). We used 63 days to estimate apparent survival to minimize underestimates of survival due to mark loss. To assess sensitivity of our survival models to encounter history length, we present daily survival estimates using encounter histories of 35–84 days.

As tree voles were only marked in the summer months (June–August), our survival inferences were limited to June–October. To provide an estimate of annual survival comparable to previous studies (Swingle et al., 2010), however, we assumed that if survival and predation risk were constant year‐round, extrapolating our 1‐day survival rate to a 1‐year period (365 days) would provide a valid comparison. To assess the assumption that predation was consistent year‐round in our study, we summarized our qualitative assessment of weasel predation for each month (Figure S1). We pooled data across years but provided separate estimates for males and females.

Although estimating mean daily survival to annual is relatively straightforward ([daily survival rate]^365^), estimating the appropriate variance around the newly rescaled parameter can prove problematic. To address this, we used the delta method to temporally rescale our estimate of variance for daily survival to annual (Powell, 2007).

Apparent survival can underestimate actual survival compared to known‐fate estimates as it is impossible to distinguish emigration from mortality within the model. We presented data on observations of known fates of marked tree voles, including observations of mortality due to predators and emigration when a marked tree vole was observed at a different nest platform than the capture location. Finally, we estimated annual apparent survival using the rate of recapture of tree voles marked with microchips in 2017 and recaptured 1‐year later in 2018.

## 3 RESULTS

We monitored 168 nest platforms using remote cameras for 670 ± 264 days; 28 cameras monitored nest platforms for 3 years, 79 monitored for 2 years, and 61 for 1 year. We monitored 34% ± 13% of nest platforms at sites (*n* = 14) in which we estimated encounter histories of marked tree voles and cameras were placed at a density of approximately one camera per 1.5 ha at those sites.

Patterns of nest platform use varied by species. For example, the mean number of detections per week was higher for tree voles and birds digging than flying squirrels, weasels, owls, or raptors. We found a higher weekly detection rate (No. of detections per monitoring‐week) for tree voles and flying squirrels compared to other species or groups (Table 1) but tree voles were detected many more times per week than flying squirrels (Figure 3a, Figure S3). Tree voles and flying squirrels were detected at all 23 sites, weasels at 18, birds digging at 16, and owls at 19. Weasels were typically detected at tree vole nests (71/82 detections) that had been occupied recently <48 hr (50/71; Figure S2).

**TABLE 1:** Summary of temporal patterns at nest platforms

Species	Proportion of monitoring‐weeks with detection	Number of monitoring‐weeks detected	Detections per week^a^	No. of sites observed	Predation rate
Tree vole	0.370	5,744	80.1 ± 107.4	23	n/a
Flying squirrel	0.446	6,915	5.5 ± 10.8	23	0.0003
Birds digging	0.017	267	31.3 ± 44.6	16	0.0037
Owl	0.006	99	3.3 ± 5.0	19	0.0103
*Small owl* ^b^	0.004	56	2.7 ± 3.1	16	0.0179
*Barred owl*	0.003	43	3.7 ± 6.7	15	0.0000
Weasel	0.005	82	3.5 ± 3.8	18	0.1000
*Short‐tailed weasel*	0.003	45	4.0 ± 4.5	14	0.1556
*Long‐tailed weasel*	0.002	37	2.5 ± 1.7	11	0.0270
Raptor (*Accipiter* sp.)	0.002	31	1.8 ± 1.2	13	0.0000

Note

Summary of different species or groups detected at 168 nest platforms monitored by remote cameras for up to 177 weeks (670 ± 264 days; $\bar{x}$ ± *SD*) at 23 young forest sites in the central Oregon Coast Range, Oregon, USA. Italics indicate species that represent a subset of a taxonomic group. Data represent weekly detections (No. of detections per week) during the sampling period in which each nest platform was monitored (*n* = 15,510 monitoring‐weeks). Predation rate is the proportion of detections resulting in an observed mortality of a red tree vole attributable to a given nest predator.

^a^ Data include only weeks in which a species or group was detected such that no 0 values were included. $\bar{x}$ ± 1 *SD*.

^b^ Northern saw‐whet owl (*n* = 20), Western screech owl (*n* = 9), Northern pygmy owl (*n* = 9), Unidentified small owl (*n* = 17).

### 3.1 Predator–prey temporal overlap models

**FIGURE 2:**
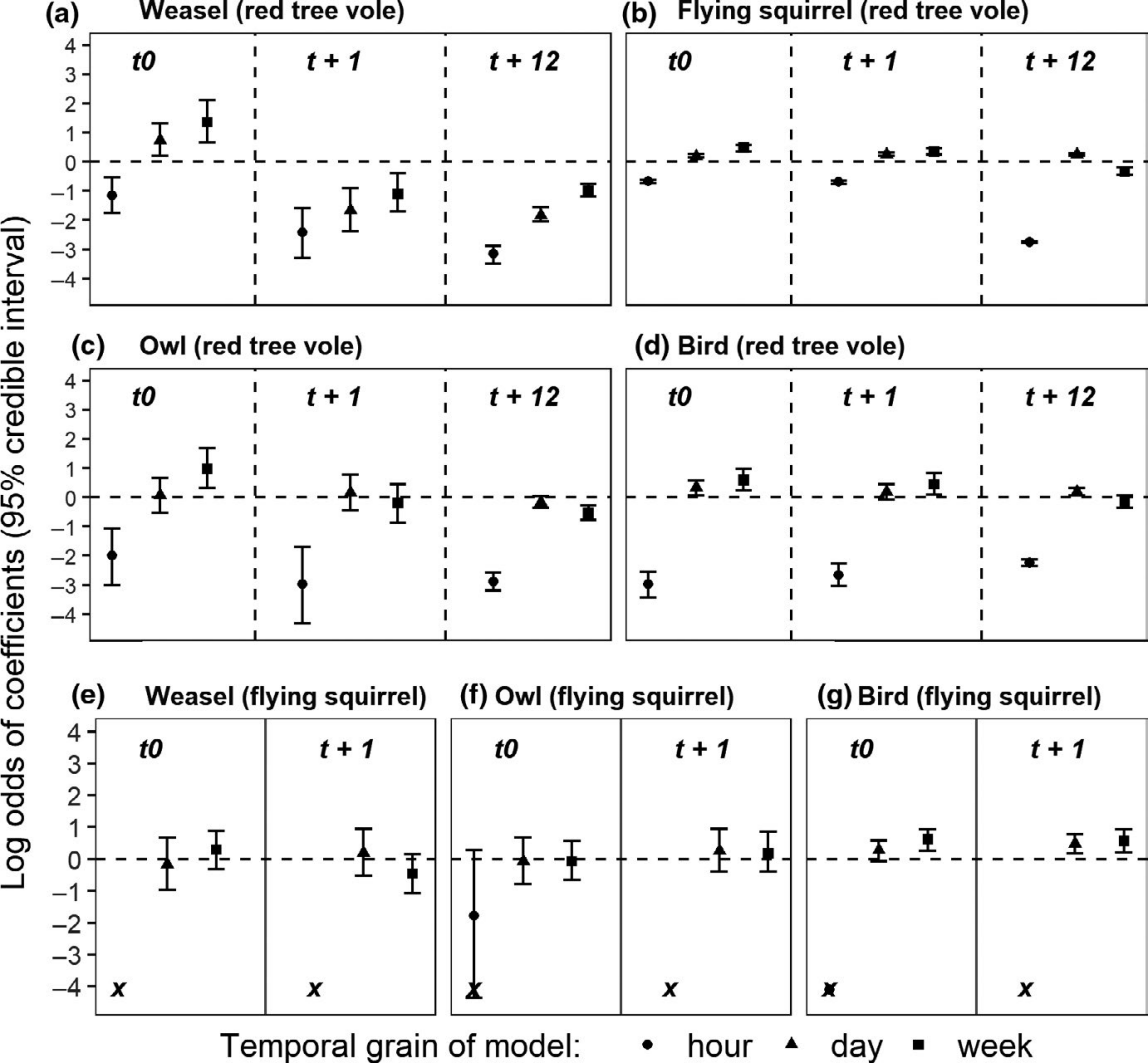
Logistic regression model results ($\bar{x}$ and 95% confidence interval) of predator–prey temporal overlap at nest platforms of four potential nest predators modeled as independent variables: Weasel, Flying squirrel, Owl, Bird: and two potential prey species as dependent variables (red tree vole, flying squirrel; shown in parentheses). Each model contains predators, two random effects, and is a unique combination of three temporal grains (bin widths) and three time lags (red tree voles; panels a, b, c, d) or three bin widths and two time lags (flying squirrels; panels e, f, g). The × in flying squirrel models indicates negative values less than four with hour as the bin width

For models of *t*0 (no lag) with 1‐day and 1‐week bin width encounter histories, detections of weasels (Figure 2a), flying squirrels (Figure 2b), owls (Figure 2c), and birds (Figure 2d) were all positively correlated with tree voles, consistent with a priori predictions. Tree voles were consistently negatively correlated with weasels in *t* + 1 and *t* + 12 models but uncorrelated with owls. Interpreting odds ratios, we observed an approximately 10‐fold decrease in odds of detecting a tree vole from *t*0 (days) to *t* + 1 (1 day after a weasel was detected), from an odds ratio of 2.1 (95% *CrI*: 1.2, 3.7) to an odds ratio of 0.19 (95% *CrI*: 0.09, 0.41). Odds of detecting a tree vole remained low 0.16 (0.13, 0.21) when modeling time steps to day 12 (*t* + 12). Tree vole detections were weakly positively correlated with flying squirrels and birds digging one time lag after these predators were detected (*t* + 1; Figure 2).

Contrary to our prediction of no interaction, flying squirrels were weakly but consistently positively correlated with birds digging (except for hour) across bin widths and time lags (Figure 2g, Figure S3) but uncorrelated with other species, only showing a weak negative correlation with weasels in the *t* + 1 weekly model (Figure 2e). Across models, we observed consistent negative correlations among dependent and independent variables for all models with 1‐hr bin widths (Figure 2).

### 3.2 Lethal and nonlethal effects of predators and temporal trends in activity

We observed 12 mortalities of tree voles at nest platforms, of which seven were attributed to short‐tailed weasels (*M. erminea*), one to a long‐tailed weasel (*M. frenata*), two to flying squirrels, one to a small owl (northern saw‐whet owl), and one to a Steller's jay (*Cyanocitta stelleri*; Table 1). Of the 12 mortalities, eight were adult tree voles and four were juveniles. In addition, we observed one flying squirrel mortality which we determined was a young animal during an annual climbing inspection, and one case where a barred owl used the nest platform as a perch to consume a brush rabbit (*Sylvilagus bachmanni*).

**FIGURE 3:**
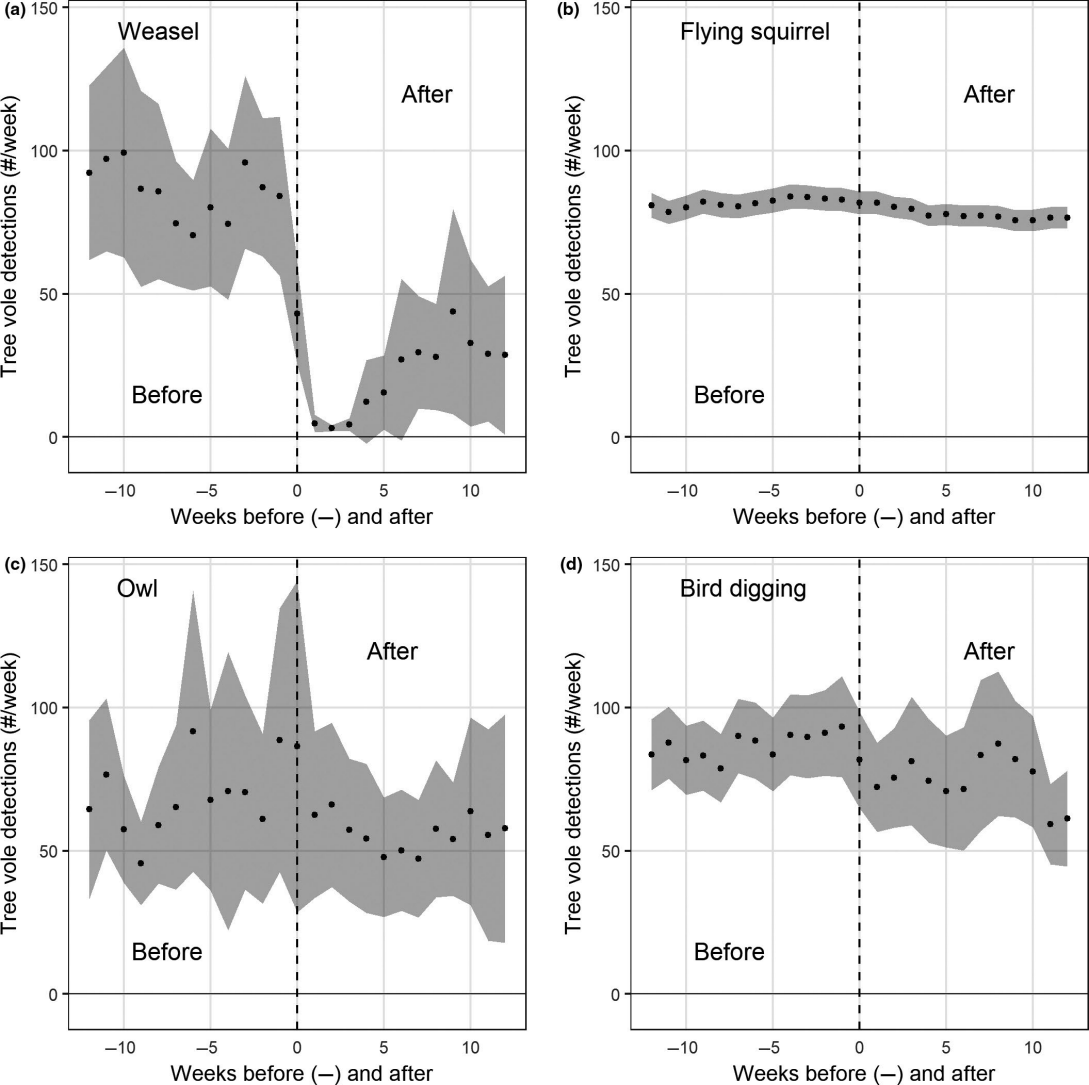
Index of tree vole activity at nest platforms before and after predator detections. Each black dot represents mean number of detections of tree voles per week (gray shading is 95% *CI*) and plots are centered on the detection of a nest predator (vertical dashed line) and include detections 12 weeks before and after predator detection. We defined bird digging as an event whereby a bird turned over nest material at the nest for >3 min

Tree vole activity (number of detections per week at nest platforms with detections) during the week prior to a weasel detection was 84.1 detections/week (95% *CI*: 56.2, 111.9) with tree voles detected at 61 nest platforms. One week following a weasel detection tree vole activity decreased to 4.7 detections/week (95% *CI*: 1.7, 7.8) with tree voles detected at only 29 nest platforms and activity remained low for at least 12 weeks postdetection (Figure 3a). Tree vole activity, on average, was constant but highly variable 12 weeks before and after a detection of owls and birds digging, and weakly negative for flying squirrels with lower variance (Figure 3).

**FIGURE 4:**
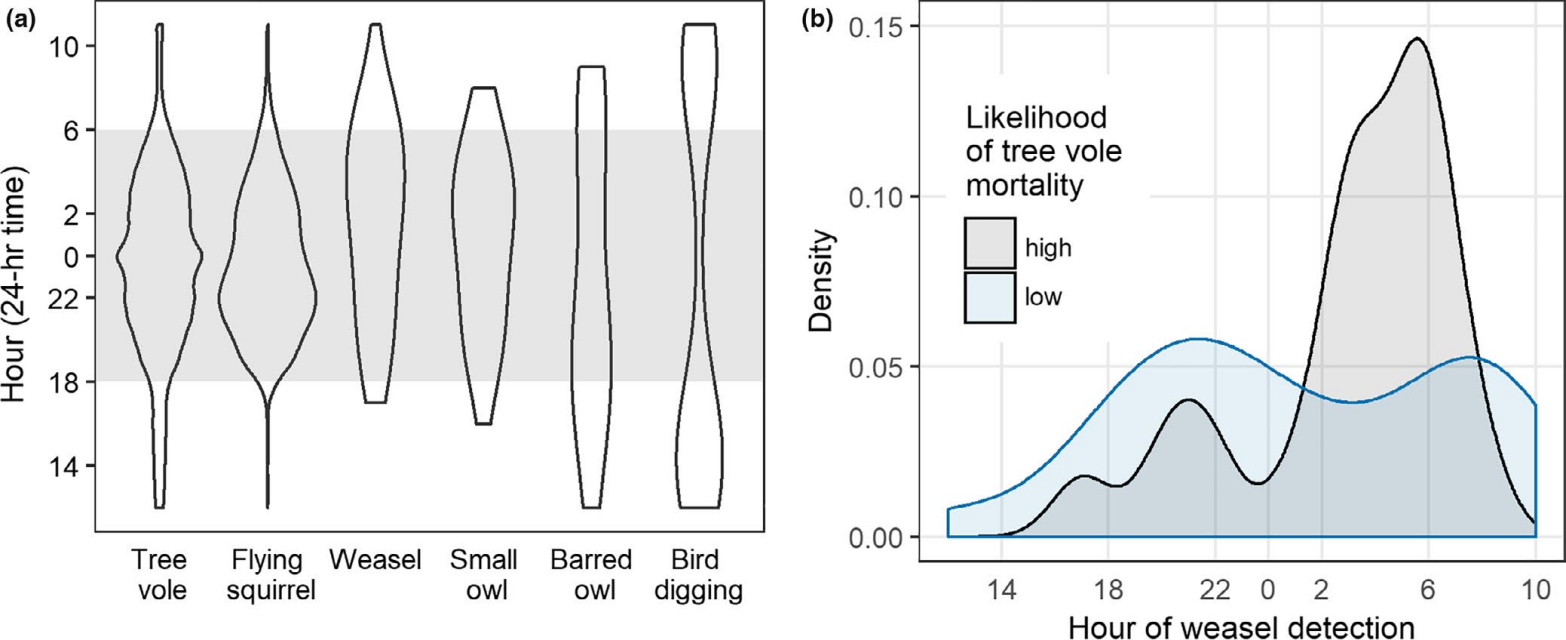
Diel activity patterns of tree voles and nest predators. Density plots (a) showing the hours in which six species or groups were detected (using hour as the bin width such that only one detection per‐hour is included) at nest platforms. Shading indicates average nocturnal period in the study area. Panel (b) shows weasel density plot split into weasel detections whereby we assigned a high and low likelihood of a tree vole mortality after a weasel was detected

Diel activity periods broadly overlapped among tree voles, flying squirrels, owls, and weasels with tree voles peaking in the middle of the night and flying squirrels in the nocturnal period before midnight (Figure 4a). Small owls and weasels showed weak positive trends in activity near dawn with barred owls arrhythmic but these species or groups had much smaller sample sizes and should be cautiously interpreted (Figure 4a, Table 1). Digging birds were active at nest platforms during the day (Figure 4a). Although weasels were detected throughout the diel period, they had a higher likelihood of preying upon a tree vole in the early morning (Figure 4b).

**FIGURE 5:**
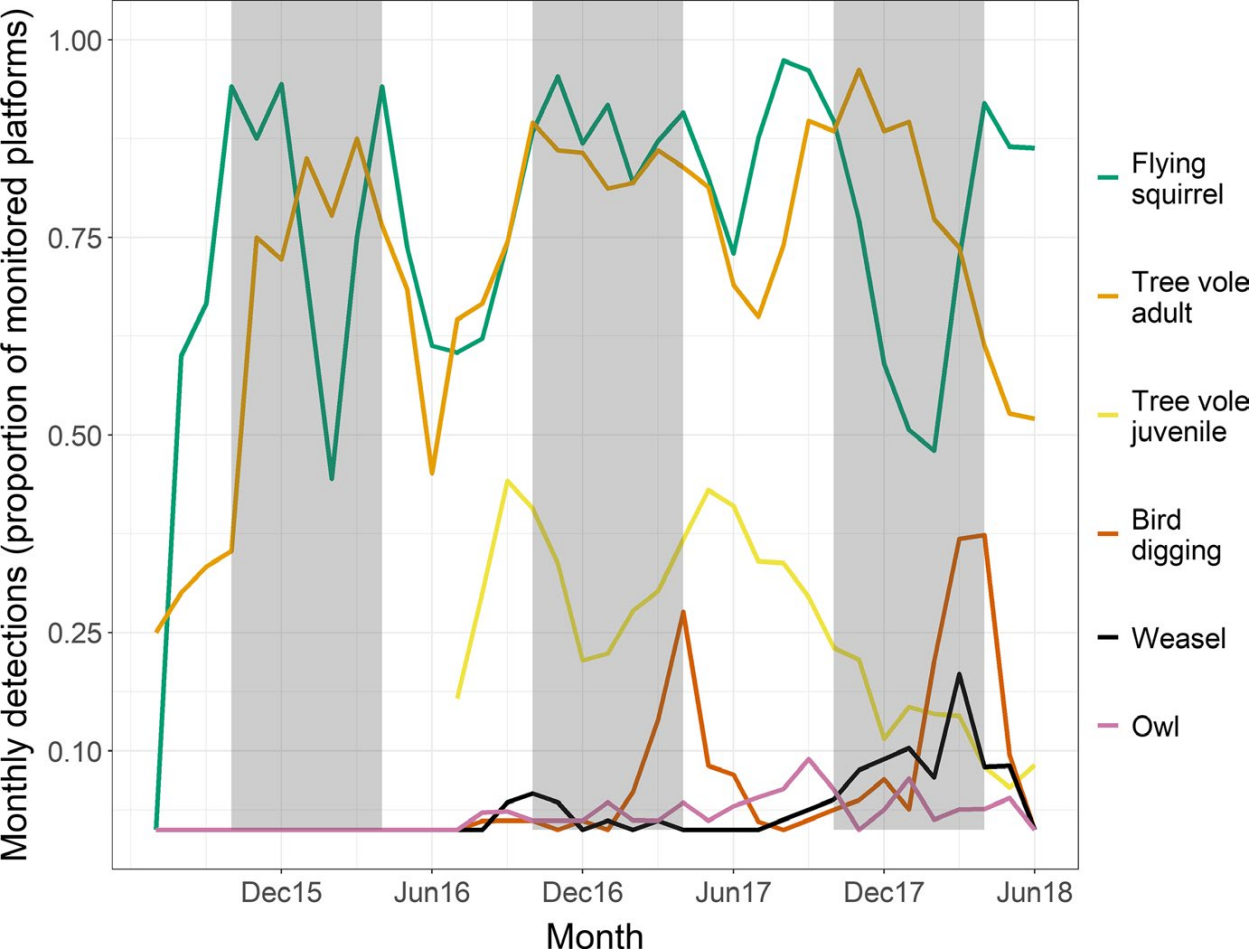
Proportion of nest platforms with detections summarized by month for several vertebrate species. We separated detections of tree voles by age class. Bird digging was an event whereby a bird turned over nest material at the nest for >3 min. Vertical gray boxes indicate the wet season in western Oregon (November 1– April 1). Note: juvenile tree voles were only assessed for photographs later than June 2016 and we truncated the data to reflect this

We observed a relatively strong pattern of birds digging late in wet seasons (February–April) of 2016 and 2017, but weaker trends for other species or groups (Figure 5). Tree voles were detected on most nest platforms with some reduction in use during the dry season, but presence of juveniles was also highest during this season in 2016 and 2017 (Figure 5). During most months, flying squirrels were detected on most nest platforms with lower number of nest platforms with a detection during February of 2016 and 2018 (Figure 5). Weasels were not detected at nest platforms until summer of 2016 and proportion of platforms with detections remained low until the wet season in late 2017 to early 2018, which was also a peak of birds digging detections (Figure 5).

### 3.3 Apparent survival of tree voles

**FIGURE 6:**
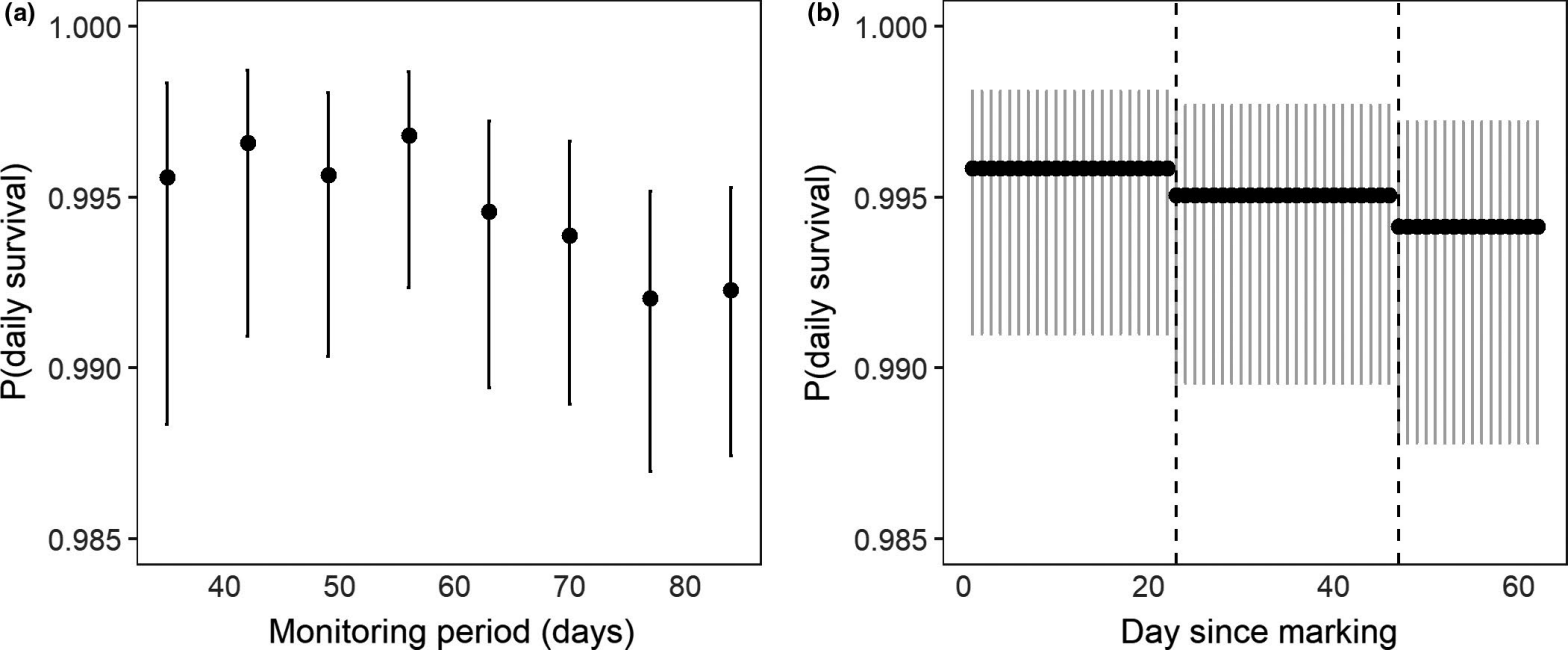
Sensitivity of survival estimates of female red tree voles to length of monitoring period (a) and daily probability of nest survival when a weasel was detected (b). The dashed line in (b) indicates the first day at which a weasel was detected at a platform inhabited by a marked tree vole

Mean daily apparent survival rate (DSR) for female tree voles was 0.9946 (*SD* = 0.011; *n* = 34) and 0.983 for male tree voles (*SD* = 0.003; *n* = 7) using a 63 days sampling period. Since survival cannot be zero, we present minimum survival in brackets. Estimated annual apparent survival was higher for females (0.14; 95% *CI*: −0.04 [0.01], 0.32) than males (0.01; 95% *CI*: −0.02 [0.01], 0.02), although 95% confidence intervals overlapped each other and zero. Daily survival was sensitive to the length of the monitoring period with a gradual decline in estimated survival in periods longer than 56 days (Figure 6a). Presence of a weasel at a nest platform decreased daily survival of tree voles throughout the marking period (Figure 6b). We observed two mortalities of marked tree voles, at 25 and 47 days after capture; both were attributed to a short‐tailed weasel. Nine of 34 females (26%) and four of seven (57%) males were observed at two nest platform with mean distances moved of 138.2 ± 68.7 m and 84.5 ± 36.4 m from initial to subsequent nest platform, respectively; none were observed at >2 nest platforms. Of 40 adult tree voles (f = 35, m = 5) captured and marked with a micro‐chip in 2017, we recaptured two in 2018 for an annual survival of 0.05.

## 4 DISCUSSION

We identified strong lethal and nonlethal effects of active, seeking predators (weasels) on arboreal rodent presence, activity, and survival at nest platforms. Arboreal rodents, primarily tree voles, were killed most frequently by weasels and their activity several weeks post weasel detection remained depressed. Secondary nest predators, thrushes, and jays probed and dug out nests in apparent pursuit of invertebrates in discarded and decaying nest materials of tree voles, and were positively correlated with flying squirrel presence, perhaps indicating that flying squirrels were attracted to nest disturbances.

Predator foraging mode can strongly influence the timing and location of where prey is killed. In our study, weasels (i.e., active, seeking predator) were the strongest nest predators whereas owls and flying squirrels were relatively weak. Similarly, Swingle et al. (2010) attributed 15 of 25 tree vole mortalities to weasels (3/25 were owls), nearly all of those were female tree voles (14/15). Owls were detected at similar rates as weasels but may simply be ineffective nest predators, unable to reliably enter or drive tree voles from their nests. Our inferences were limited to nest platforms in young forests and did not extend to foraging tree voles or to the old forests in which strong relationships between tree voles and one of their main predators, the northern spotted owl, have been established (Forsman, Anthony, Charles Meslow, et al., 2004; Forsman, Anthony, & Zabel, 2004; Forsman, Swingle, Davis, Biswell, & Andrews, 2016). Nonetheless, our results provide evidence that tree voles face strong pressure from weasels but not owls at their nests.

Although foraging mode may have differed, diel activity patterns of predators broadly overlapped those of tree voles except for diurnally active digging birds. Weasels can be active throughout the day (Linnell, Epps, Forsman, & Zielinski, 2017) but appeared to be more effective at capturing tree voles in the morning hours (Figure 4b), a pattern similar to least weasels (*M. nivalis nivalis*) which were more active at sunset but captured most *Microtus* field voles in the morning (Sundell, Norrdahl, Korpimäki, & Hanski, 2000). Determining if this reflects a temporal vulnerability of prey will require additional studies, including where and how weasels use cues to locate arboreal prey.

Weasels can cue into rodent scent when hunting, and scent can accumulate at or near small rodent nests and is hypothesized to increase predation risk there (Sharpe & Millar, 1990; Ylönen, Sundell, Tiilikainen, Eccard, & Horne, 2003). Female tree voles may be especially at risk of mortality at their nests because of high nest fidelity and that their nests are large and may be conspicuous to predators (0.06 m^3^; Swingle, 2005; Sharpe & Millar, 1990). Alternatively, large tree vole nests typically contain multiple tunnels and chambers, providing shelter and escape routes from predators (Maser, 1966). The relative predation risk associated with long‐term habitation remains unknown but the higher visitation rates by weasels after year 3 in our study indicates a potential response of these predators to prolonged occupancy of nests by tree voles. Whether this was due to environmental cues such as scent accumulation or to numerical response of the predators is unknown but indicates predation risk can potentially limit long‐term population growth of tree voles regardless of an increase in resource availability (i.e., new nests).

Predators can cause prey to avoid areas with high resource densities and avoidance behaviors can have a greater effect on prey population densities than consumption (Preisser et al., 2005). In the case of weasels in our study, we observed potentially lethal effects followed by reduction of activity (a nonlethal effect), perhaps due to nest abandonment by tree voles for up to several weeks post weasel detection. Most weasel detections occurred toward the end of our study, limiting our inferences with regards to longer term nonlethal avoidance. But given the limited nest substrate availability in young forests, removal of even several productive nests through predation followed by nonlethal avoidance could cause a substantial limit on tree vole populations.

Escape tactics of prey are most effective against their most lethal predators, and for most voles, this includes weasels and predatory birds, and these interactions are frequently mediated by structural habitat (Jędrzejewski et al., 1993; Sundell & Ylönen, 2008). For example, forest‐dwelling bank voles (*Myodes glareolus*) were more likely to escape vertically from least weasels by climbing trees (Mäkeläinen et al., 2014). Anti‐predatory behaviors, including escape tactics, along with habitat‐dependent prey densities are hypothesized to dampen population oscillations of bank voles relative to meadow‐dwelling voles (Koivisto, Huitu, Sundell, & Korpimäki, 2008). Tree voles may exhibit similar anti‐predatory habitat selection (almost exclusive arboreal nesting and foraging) and behaviors (including rapid escape from nests by free‐fall leap) that may reduce predation risk from primarily terrestrial weasels (Forsman, Swingle, & Hatch, 2009). In addition, the typically low numbers of weasels' main prey, terrestrial voles, in closed‐canopy young forests (Gomez & Anthony, 1998) may limit predator switching (Sundell & Ylönen, 2008), contributing to higher survival. Arboreal anti‐predatory behaviors or habitat‐dependent prey densities may contribute to high survival of tree voles relative to terrestrial voles (Swingle et al., 2010) but whether, as hypothesized for bank voles, this contributes to more stable populations remains uncertain.

Specialization of predators can determine their functional response to numerical increases in their prey (Sundell et al., 2000). Short‐tailed weasels in our study are similar‐sized to least weasels which show a type II functional response (rapid initial increased predation rate with prey density indicating a high degree of specialization) to higher densities of field voles in low prey‐diversity boreal ecosystems (Sundell et al., 2000). Temperate forests typically have higher diversity of prey and weasels seem unlikely to exhibit a type II functional response to tree voles, particularly because tree voles do not reach sufficiently high densities to elicit such a response and functional response of weasels may be more similar to a generalist predator (type III) than a specialist (type II) with regards to tree voles (Sundell et al., 2000).

Homogeneity of nest platform placement could have provided a visual cue to avian nest predators (Santisteban, Sieving, & Avery, 2002) although vertical placement within the live tree canopy broadly represented height of natural nest substrates found in young forests (Linnell et al., 2018; Swingle, 2005). In contrast, older forests have much higher heterogeneity in tree height and natural substrates for tree vole nests vary in type (Lesmeister & Swingle, 2017), location within the canopy, and may be more numerous (Swingle, 2005). Moreover, higher nests in a more heterogeneous environment may disperse scent, decoupling cues from terrestrial predators. These characteristics of tree vole nests in old forest—more numerous nest substrates, varied height, size, and substrate—may make those nests more difficult to locate for predators using visual or olfactory cues.

Varying the bin width of our continuously collected remote camera data obscured biological relationships and, in some cases led to biologically counter‐intuitive results. At our finest bin width (1‐hr), all correlations were negative, even for weasels which were typically detected within 48 hr of a tree vole (50/82 detections). Moreover, weasels clearly exhibited the strongest negative lethal and nonlethal effects on tree voles but correlations were indistinguishable from other species at the 1‐hr bin width. Interpretation of predator–prey interactions depended on selecting the relevant temporal grain size and we urge thorough examination of continuous data, such as counts of activity or direct observations of predator–prey interactions (e.g., mortality), to support inferences made from any given bin width.

Our models were likely inadequate at detecting weak nonlethal interactions occurring over longer time periods, such as potential interference competition between flying squirrels and tree voles. Flying squirrels were ubiquitous but weak lethal predators of tree voles at nests killing them at a rate of 0.03% (*n* = 2 observed mortalities) compared to the 10% (*n* = 8 observed mortalities) predation rate of weasels (Table 1). Nonetheless, we observed a weak decrease in tree vole activity up to several weeks after detection of a flying squirrel (Figure 3b) providing circumstantial evidence that flying squirrels exhibit weak interference competition with tree voles, although further evidence would be needed to corroborate this observation.

For female tree voles, annual survival (0.14; 95% *CI*: [0.01], 0.32) was similar to that of a radio‐telemetry study (0.12; 95% *CI*: 0.01, 0.31) but for males, our estimate of 0.01 (95% *CI*: [0.01], 0.02) was much lower (0.33; 95% *CI*: 0.07, 0.76; Swingle et al., 2010). The high re‐sighting rates of females, mainly attributable to high nest site fidelity, contributed to more precise estimates of survival although our sampling period was truncated due to external marks fading quickly. Increasing the longevity of artificial marks would greatly improve our estimates, especially for females. Using external marks to visually recapture marked tree voles using multiple remote cameras provided similar survival estimates to radio‐telemetry for female tree voles and appears appropriate for monitoring nests of high nest fidelity animals but would benefit from longer‐lasting external marks.

Tree voles responded with strong population growth in 2016 1 year after addition of nest platforms (Linnell et al., 2018). Herein, we demonstrated that predator activity increased over a 3‐year monitoring period and had strong lethal and non lethal effects on individual tree voles at nest platforms. The success or failure of nest‐box and nest platform to increase prey population size may ultimately depend on longer term predator–prey dynamics (Sonerud, 1985). Our evidence indicates that predators, particularly weasels, can exert strong acute effects on tree vole activity at nests in young forests. Determining whether predation pressure, resulting in mortality and reduced occupancy of already scarce nest sites, contributes substantially to patterns of long‐term local instability of tree vole populations in young forests will require longer term monitoring.

## CONFLICT OF INTEREST

The authors declare no competing interests.

## AUTHOR CONTRIBUTIONS

**Mark A. Linnell:** Conceptualization (equal); Data curation (lead); Formal analysis (lead); Funding acquisition (supporting); Investigation (equal); Methodology (equal); Project administration (supporting); Resources (equal); Software (lead); Supervision (equal); Validation (lead); Visualization (lead); Writing‐original draft (lead); Writing‐review & editing (equal). **Damon B. Lesmeister:** Conceptualization (equal); Data curation (supporting); Formal analysis (supporting); Funding acquisition (lead); Investigation (supporting); Methodology (equal); Project administration (equal); Resources (equal); Software (supporting); Supervision (equal); Validation (supporting); Visualization (supporting); Writing‐original draft (supporting); Writing‐review & editing (equal).

## PERMITS

Capture and handling methods were approved by the U.S. Forest Service Institutional Animal Care and Use permit #2016‐009 and Oregon Department of Fish and Wildlife Scientific Taking Permit 041‐18.

## Supporting information

Figure S1

Figure S2

Figure S3

Supplementary Material

## Data Availability

Data are available at the Dryad Digital Repository https://doi.org/10.5061/dryad.cjsxksn3d.

## APPENDIX A

### Assigning qualitative likelihood that weasels preyed upon tree voles

We present additional data on mortality of tree voles attributable to weasels including assessing whether we may have underestimated the number of mortalities. For example, predation could have gone undetected due to our photo sampling schedule (i.e., if a mortality occurred during quiet period of the remote camera) or due to weasels accessing the nest platforms outside of the remote camera field of view (e.g., from the bottom). We used these data to evaluate our assumptions of consistent year‐round predation risk from weasels and to compare annual rates of likely predation (Figure S1).

We first qualitatively evaluated likelihood of tree vole mortality due to weasel. We reviewed photographic sequences up to several weeks before and after weasel detections at nest platforms that contained tree vole nest sign (*n* = 71) and assigned a likelihood of predation as observed, high, moderate, low. We added an additional category whereby no predation was possible (none) and that was defined as when a weasel was detected at a nest platform where a tree vole was not present (no detection for up to 48 hr prior to the weasel being detected).

After scoring each weasel detection for likelihood, we compared our qualitative categories using indices of tree vole activity. First, we estimated whether a tree vole was likely present when a weasel was also detected using number of detections 48 hr prior to the weasel detection and time difference in last known tree vole detection (Figure S2a,b). Second, we estimated whether a tree vole was likely preyed upon by estimating activity of tree voles after the weasel detection (Figure S2c).

We further assessed whether a tree vole was present up to 48 hr after detection of the weasel and present the average time, excluding records when a tree vole was not detected in the 48‐hr period. The proportion of nest platforms whereby a tree vole was detected after the weasel detection by likelihood category was as follows: none = 0, low = 0.55, moderate = 0.50, high = 0.14, and observed = 0.43. The low value of the “high” likelihood category indicates that our qualitative assessment likely depended heavily on assessing whether a tree vole was detected after the weasel detection. In addition, we summarize index of weekly activity for flying squirrels before and after another species was detected (Figure S3).
