# Supplementary figures and images for: Predator–prey interactions in the canopy
Source: Ecol Evol. 2020 Jul 29;10(16):8610–22. doi: 10.1002/ece3.6518 (PMC7452817; doi:10.1002/ece3.6518)

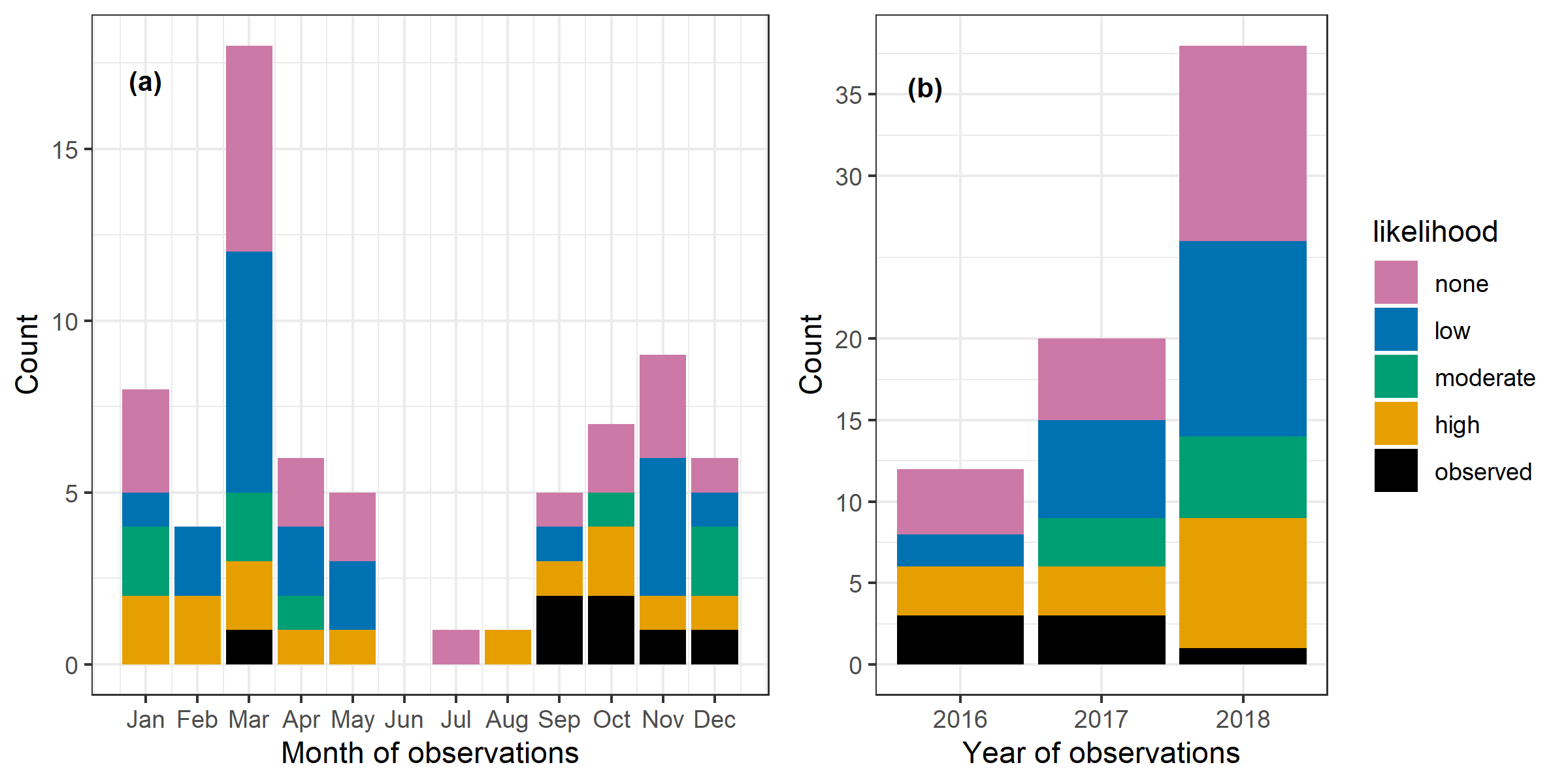

Supplement: Supplementary file 1 — Figure S1 [file ECE3-10-8610-s001.tiff]

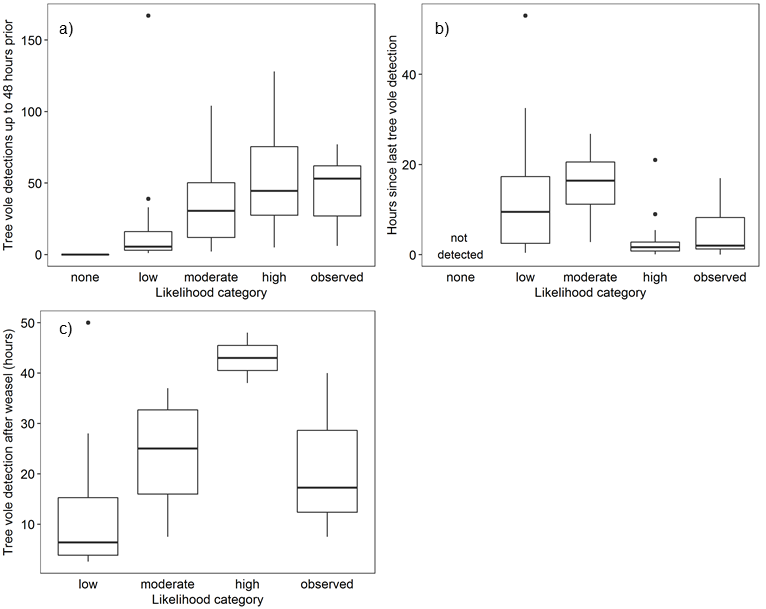

Supplement: Supplementary file 2 — Figure S2 [file ECE3-10-8610-s002.png]

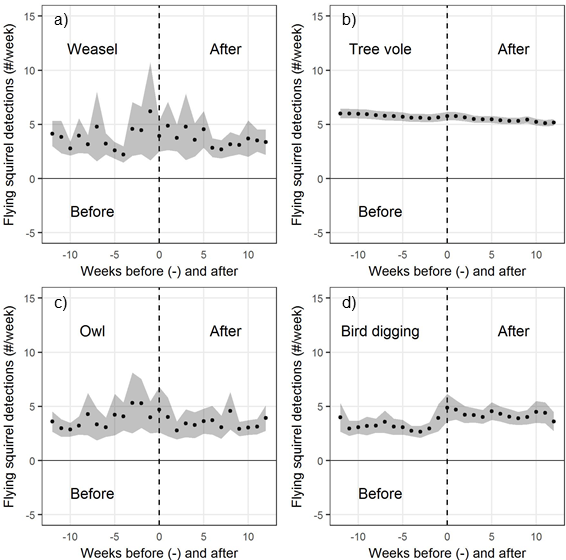

Supplement: Supplementary file 3 — Figure S3 [file ECE3-10-8610-s003.png]
