# Supplementary material for: Predator–prey interactions in the canopy
Source: Ecol Evol. 2020 Jul 29;10(16):8610–22. doi: 10.1002/ece3.6518 (PMC7452817; doi:10.1002/ece3.6518)
Supplement: Supplementary file 4 — Supplementary Material [file ECE3-10-8610-s004.docx]

Figure S1. Assigned likelihood of predation by a weasel at a nest platform by year and by month. We assigned qualitative likelihood of predation by reviewing sequences of photographs to assess presence and activity of tree voles before and after a weasel detection. “Observed” indicates sequences in which we observed a dead tree vole in the presence of a weasel and indicates a confirmed predation event. Sample sizes were none (tree vole not detected; n = 21), low (n = 20), moderate (n = 8), high (n = 14), and observed (n = 8).

Figure S2. We used several metrics to support our qualitative likelihood assessment that a tree vole was preyed upon by a weasel. All panels indicate time relative to a weasel detection at a tree nest occupied within the previous 48 hours (panels a and b or 72 hours: panel c). The x-axis is the assigned likelihood category that a weasel detection resulted in a tree vole mortality with “observed” indicating that a tree vole was observed dead on the top of the nest platform, none (tree vole not detected up to 48 hours prior) and the other categories our assigned likelihood from our qualitative assessment. Panel a indicates the number of tree vole detections up to 48 hours prior to a weasel detection at a platform (y-axis). Sample sizes of number of monitored platforms for each category in panel a: none (n = 21), low (n = 20), moderate (n = 8), high (n = 14), and observed (n = 8). Panel b indicates the number of hours since the most recent tree vole detection up to 48 hours prior to the weasel detection. Panel c is the time lag in hours in which a tree vole was detected after a weasel detection. Sample sizes and proportions are as follows for each of our qualitative likelihood categories in panel c: low (n = 11; 0.55), moderate (n = 4; 0.5), high (n = 2; 0.14), and observed (n = 3; 0.43).

Figure S3. Index of flying squirrel activity (# detections per week) before and after detections of other species. The mean is indicated at each time step (dot) and the 95% confidence intervals shown as gray shading. We defined bird digging as an event whereby a bird turned over nest material at the nest for >3 minutes. We omitted zero values for #/week such that the sample for each week and species combination only includes nests where a flying squirrel was detected at least once.
